# Extracellular Vesicles—Oral Therapeutics of the Future

**DOI:** 10.3390/ijms23147554

**Published:** 2022-07-07

**Authors:** Martyna Cieślik, Katarzyna Nazimek, Krzysztof Bryniarski

**Affiliations:** Department of Immunology, Jagiellonian University Medical College, 18 Czysta St., 31-121 Krakow, Poland; martyna.cieslik@uj.edu.pl (M.C.); krzysztof.bryniarski@uj.edu.pl (K.B.)

**Keywords:** exosomes, extracellular vesicles, nanovesicles, nanoparticles, immune modulation, oral treatment

## Abstract

Considered an artifact just after discovery, the possibility of oral delivery of extracellular vesicles (EVs) and their functional cargos has recently gained much research attention. EVs from various sources, including edible plants, milk, bacteria and mammalian cells, have emerged as a platform for miRNA and drug delivery that seem to induce the expected immune effects locally and in distant tissues after oral administration. Such a possibility greatly expands the clinical applicability of EVs. The present review summarizes research findings that either support or deny the biological/therapeutical activity of orally administered EVs and their role in cross-species and cross-kingdom signaling.

## 1. Introduction

Recently, extracellular vesicles (EVs) that may contain microRNA (miRNA) molecules together with freely circulating miRNAs have attracted research attention as promising candidates for various preventive and therapeutic interventions [1,2]. Therefore, a growing number of studies have evaluated the therapeutic potential of EVs and miRNAs, the possible maneuvers to increase their effectiveness in vivo [3,4], and their biodistribution, pharmacokinetics and pharmacodynamics [5,6,7,8]. In this regard, selection of the most efficient route of administration is a crucial step that determines further properties of EV- and miRNA-based therapeutics [9].

**Figure 1:**
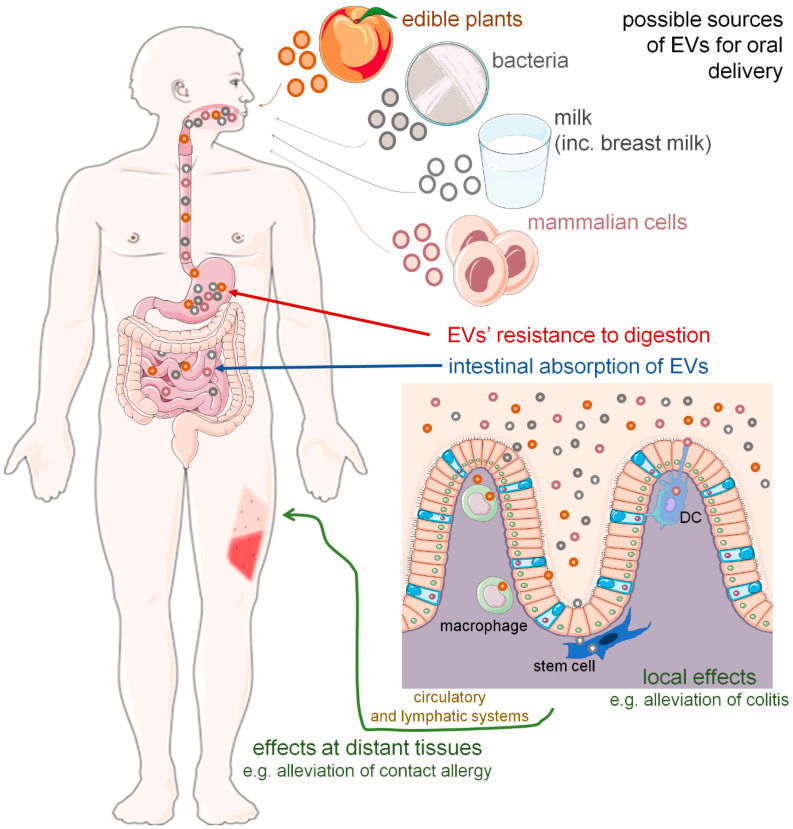
Oral route of extracellular vesicle (EV) delivery could induce local and distant biological effects. Orally administered EVs originating from various sources were found to be resistant to harsh conditions, including acidic and alkaline pH, and thus not digested but instead absorbed in intestines. Then, EVs appear to target intestinal macrophages, dendritic cells (DC) and stem cells to induce expected modulatory/therapeutic effects either locally, in the digestive tract, or at the periphery, when transported in the blood plasma and/or lymph. Some of the icons were adopted from smart.servier.com (accessed on 12 June 2022) and used in compliance with the terms of the Creative Commons Attribution 3.0 Unported License.

Postulated cross-species and cross-kingdom transmission of small non-coding RNAs, particularly miRNAs [10,11], via the oral route remains controversial. However, it has been speculated that lipids support the intestinal transmission of miRNAs [12]. This opened up another research direction, namely studies on EV-based transfer and functionality after oral delivery. As herein discussed, EVs from various donor organisms have recently emerged as a platform for miRNA and drug delivery via the oral route and seem to induce expected immune effects locally and in distant tissues (Figure 1).

## 2. Plant-Derived EVs in Cross-Kingdom Delivery—Truth or Imagination?

Recently, growing evidence has suggested possible therapeutic applications for nanoparticles derived from edible plants [13,14,15], especially when administered orally to induce immunomodulation. However, their entry pathways and activities are still debated. Studies on the Caco-2 cell line have suggested that apple-derived nanoparticles may be internalized by intestinal epithelial cells [16]. Additionally, other target cells for orally administered nanoparticles have been proposed, including intestinal macrophages [17,18], dendritic cells (DCs) [19] and mesenchymal stem cells [20] (Figure 1).

Orally delivered grapefruit-derived nanoparticles were shown to ameliorate dextran sulfate sodium (DSS)-induced colitis in mice by decreasing the expression of chemokine mRNAs, which reduces the recruitment of inflammatory T lymphocytes and monocytes. Intestinal macrophages were proposed to absorb these nanoparticles, likely through both micropinocytosis and clathrin-dependent pathways, which enhances the expression of anti-inflammatory factors, including IL-10 [17]. Similar alleviation of colitis was observed in mice with orally administered broccoli-derived nanoparticles that appear to target mesenteric lymph nodes and intestinal DCs, in which a reduction in TNF-α, IFN-γ and IL-17A, as well as overexpression of IL-10 was observed [19].

Other research described a protective effect against severe DSS-induced colitis in mice administered with nanoparticles derived from grapes by gavage that were found to be micropinocytosed by intestinal stem cells, which enhanced their proliferation in both the small intestine and colon [20]. This phenomenon may be of great importance in homeostasis maintenance because self-renewal of the intestinal epithelium is necessary to support proper integrity [21]. Grape-derived nanoparticles were also found to be resistant to the artificially created environment of the stomach and to induce overexpression of the Lgr5 receptor in rats’ intestinal stem cells [22], which suggests increased proliferation [23]. This provides further evidence of the effectiveness of an approach based on the use of plant-derived EVs in remodeling intestinal cells and exploiting the therapeutic effects of this process.

Nanoparticles obtained from acerola juice appear to conjugate with miRNA molecules, which makes the latter more stable in RNase, acidic and alkaline environments when compared with miRNA conjugated with human breast milk EVs. Acerola nanoparticles seem to be taken up by intestinal macrophages and then distributed via circulation to different organs, predominantly to the intestine, liver and bladder. Interestingly, some were also detected in mouse brains, indicating the possibility of using this route of administration to deliver miRNAs to the central nervous system [18].

Conversely, orally administered ginger-derived nanoparticles were primarily expressed in the liver and mesenteric lymph nodes but not in other organs in mice. The authors suggested that hepatocytes incorporated the nanoparticles using specific endocytosis pathways, leading to an increase in the expression of detoxifying/antioxidant genes, such as HO-1, NQO1, GCLC and GCLM, which may explain their demonstrated protective effect on alcohol-induced liver damage [24].

However, the role of plant-derived nanoparticles in cross-kingdom delivery remains controversial. Some studies failed to detect plant-enriched miRNAs in the plasma of different animals after plant product ingestion [25], while others demonstrated the presence of plant-derived miRNAs in mouse [26] and human sera [27]. Furthermore, some studies suggest the possibility of regulating mammalian cell transcripts by exogenous, diet-derived miRNAs [28,29,30,31]. This leads to the idea of supplementing food with therapeutic miRNAs. However, currently, the scientific community is far from being convinced of this idea [32,33], especially as some studies indicate an insufficient passage of exogenous plant miRNA to mouse sera after oral feeding [26]. On the other hand, miRNAs from edible plants may modulate gut microbiota [34], and such possible changes would in turn impact the body’s functioning. However, after absorption from the gastrointestinal tract, plant miRNAs may potentially target immune cells, and thus could play an important role in immune regulation [35]. Accordingly, EVs appear to be the most efficient vehicles for the oral delivery of plant miRNAs.

Interestingly, except from extrinsic miRNA, plant-derived nanoparticles could be conjugated with drugs, e.g., methotrexate. In DSS-induced colitis, an orally delivered methotrexate–grapefruit nanoparticle complex showed a pronounced immunomodulatory effect with reduced drug-dependent cytotoxicity [17]. Altogether, plant-derived nanoparticles could be considered promising candidates for the oral delivery of various therapeutics. On the other hand, EVs are also considered to be interesting carriers for favorable food additives, improving the nutritional value of food to increase their stability and bioavailability [36].

## 3. Bacterial EVs—Another Source for Consideration?

Current knowledge emphasizes the important immunomodulatory impact of microbiota, which may also be mediated by microbial EVs, formerly called outer membrane vesicles (OMVs). One of the most abundant species in human gut microbiota, *Akkermansia muciniphila*, acts as a “self-probiotic”, and its presence is inversely related to metabolic inflammatory disorders, including obesity and type 2 diabetes [37]. Interestingly, both *A. muciniphila* and its EVs, administered orally by gavage, reduced body weight gain in mice fed with a high-fat diet, which was associated with a decrease in fat tissue weight and the total cholesterol concentration. These observations were confirmed by significantly reduced expression of genes for TLR-4, TNF-α and IL-6 in epidydimal adipose tissue or/and in the colon. In contrast, PPAR-γ and PPAR-α mRNA levels increased [38]. Orally delivered *A. muciniphila* and its EVs also enhanced the expression of the IL-10 gene in the colon of healthy mice [39]. Furthermore, reduced permeability of the intestinal barrier through the regulation of tight junctions was observed in obese mice as well as in mice with type 2 diabetes after administration of *A. muciniphila*-derived EVs [38,40], which was also confirmed in an in vitro study [41]. However, four-week administration of this bacteria and their EVs to mice by oral gavage also affected serotonergic gene expression, both in the colon and hippocampus, indicating that bacterial EVs had an impact on the gut–brain axis [39]. Furthermore, it has been suggested that the possible penetration of the brain by orally administered *Paenalcaligenes hominis*-derived EVs through the blood and the vagus nerve may promote cognitive impairment in mice [42]. Metagenomic data revealed significant changes in stool EVs’ composition caused by DSS-induced inflammatory bowel disease (IBD), affecting *A. muciniphila*- and *Bacteroides acidifaciens*-derived EVs in particular. Moreover, oral application of *A. muciniphila*-derived EVs triggered a protective effect against IBD, which was manifested in a reduction in weight loss and colon inflammation [43]. EVs derived from a probiotic strain of *Escherichia coli* Nissle 1917, administered to mice by esophageal catheter, ameliorated inflammation and clinical symptoms of DSS-induced colitis [44]. Similar effects were induced by *Lactobacillus rhamnosus*-derived EVs by regulating murine gut microbiota [45]. It has been reported that *Helicobacter pylori*-derived EVs delivered intragastrically to mice may modulate both innate and adaptive immune responses using a NOD1-related pathway. Interestingly, bacterial EVs seem to be necessary for the delivery of NOD1-stimulating peptidoglycan to non-phagocytic intestinal epithelial cells [46].

Bacterial-derived EVs appear to be candidates for mucosal vaccines causing immune protection against specific pathogens [47,48,49]. Accordingly, massive production of both intestinal sIgA as well as serum IgG antibodies against EV-transmitted antigens was noted in mice that were orally immunized with three doses of chitosan-coated *Campylobacter jejuni*-derived EVs together with the induction of a specific cell-mediated immune response. Furthermore, after a subsequent intragastric challenge with *C. jejuni*, a significant reduction in the bacterial load and only slight histological changes in the cecal tissue was observed in immunized animals [47]. Similarly, four intragastric administrations of *H. pylori*-derived EVs with a cholera toxin as an adjuvant protected mice from *H. pylori* infection by activating the production of antibodies specific for EVs’ outer membrane protein [49]. Another example of a mucosal vaccine is described in studies on EVs derived from *Vibrio cholerae*. Immunization with EVs caused a long-lasting increase in antibody titers in serum depending on the route of administration. Intragastric vesicle delivery caused particularly robust responses in IgG1 and IgG2. Interestingly, the offspring of orally immunized mice were also protected from infection with *V. cholerae* [48].

## 4. “Milky Way”

Currently, growing evidence suggests that EVs at least partly mediate the immunomodulatory functions of dietary and breast milk [50,51]. The latter was shown to contain EVs that inhibited IL-2 production by peripheral blood mononuclear cells (PBMCs) and promoted activation of Foxp3^+^CD4^+^CD25^+^ regulatory T cells [52]. This appears to be mediated by immune-related miRNAs and pre-miRNAs, found in EVs isolated from the breast milk of various mammals [53,54,55], that are more abundant in early milk (especially colostrum) [54] and are evolutionarily conserved [56]. Additionally, miRNA composition seems to differ between EVs and supernatant fractions from ultracentrifuged milk [57]. Moreover, milk EVs may contain miRNAs of plant origin [58]. Studies with piglet serum revealed the time-dependent presence of some miRNAs from the previously administered milk, indicating their absorption from the gastrointestinal tract [57]. However, other studies described the possibility of postprandial transfer of milk/colostrum EVs’ membrane proteins, but not their miRNA cargo, into the bloodstream [59]. Similarly, some studies deny the transfer of dairy milk-derived miRNAs into adult human circulation [60]. On the other hand, Sadri et al. demonstrated in pregnant mice that orally delivered bovine milk EVs and their miRNA cargos may cross the placenta and accumulate in embryos, which may promote survival [61].

So far, oral delivery of bovine milk EVs to rodents was shown to not interfere with animal wellness [62,63]. However, slight changes in gene expression in muscles were noted in mice orally administered with milk-derived EVs [62], even though such EVs seem to transfer less efficiently from the gastrointestinal tract to the muscles compared with other tissues [62,64]. On the other hand, EV-depleted bovine milk administration resulted in an increase in muscle anabolism and in significant changes in the expression of various genes in rats’ skeletal muscles [65]. Although the underlying mechanism remains unknown, it could involve a potential influence on the gut microbiota of animals fed with such milk [65], especially changes in the microbial composition in mouse intestines following oral administration of milk-derived EVs, as has already been reported [66,67], and this is potentially related to an increase in the cecum size [68]. Orally delivered, bovine colostrum-derived EVs were shown to rebalance the gut microbiota disturbed by osteoporosis in mice, which correlated with the inhibition of bone resorption as well as with an improvement in bone remodeling [69]. Interestingly, EVs derived from milk could have an impact on the elongation of bones. An increase in mineral density and the average length of the tibia was noted in rats orally administered with bovine milk-derived EVs [63].

Similar to the studies about plant-derived EVs, in the case of per os administration of human and bovine milk-derived EVs, the alleviation of symptoms of DSS-induced colitis has also been described in mice and included earlier weight gain and less pronounced shortening of the colon together with downregulated expression of genes for proinflammatory cytokines, such as TNF-α and IL-6, and increased levels of TGF-β1 protein in the colon [70]. In fact, TGF-β1 is carried by some EVs [71,72], including those derived from milk [70,73], which could transfer this cytokine to tissues. The therapeutic effect of EVs may also be caused by enclosed miRNAs, including miRNA-375 and miRNA-320 [70]. Bovine milk EVs, administered by oral gavage to the small intestine, may have anti-inflammatory properties in ulcerative colitis in mice, expressed macroscopically, inter alia, through an improvement in both the appearance of ileal and colonic segments and the consistency of stool samples [74]. Conversely, mice treated with EV-depleted bovine milk develop exacerbated symptoms of IBD, associated with a decreased level of miRNA-200a-3p, abundant in bovine milk-derived EVs, and increased serum concentrations of CXCL9 [75]. The protective effect of orally delivered porcine milk EVs was reported in a study on toxin-induced damage of the small intestine in mice, which showed reduced levels of apoptosis-related caspases and the p53 protein in the jejunum tissue, a mitigated toxin-induced decrease in the expression of genes associated with cell proliferation (β-catenin and cyclin D1), as well as overexpression of genes (and related proteins) associated with intestinal tight junctions [76]. In addition, intragastric delivery of porcine milk EVs to healthy mice stimulates the proliferation of intestinal epithelial cells [77]. Similarly, human milk-derived EVs may exert beneficial effects on the intestines by protecting intestinal stem cells from oxidative stress [78]. Moreover, rat milk EVs also promoted intestinal epithelial cell growth and intestinal stem cell activity in vitro [79]. Altogether, these findings suggest the important role of milk EVs and their miRNAs in the maintenance of proper intestinal functions.

Milk EVs appear to be a promising treatment for some autoimmune disorders, as shown in the arthritis models in mice [80]. Arntz et al. noted a dose-dependent attenuation of collagen-induced arthritis after oral administration of cow’s milk-derived EVs added to drinking water one week before immunization with collagen. A similar improvement, expressed as a dose-dependent reduction in ankle joint swelling, in bone marrow cellularity and cartilage depletion, was observed after EV delivery by gavage to IL-1Ra^−/−^ mice, which spontaneously developed polyarticular arthritis [80].

Furthermore, milk EVs could be loaded with chemotherapeutics and other drugs [81]. Agrawal et al. described that paclitaxel-loaded bovine milk-derived EVs efficiently inhibit tumor growth in the model of subcutaneous lung cancer in mice. Interestingly, a paclitaxel–EV complex was two times more effective after oral than intraperitoneal administration [82]. Moreover, EVs isolated from mid-lactation or commercial bovine milk reduce the primary tumor burden after oral administration to mice with colorectal or breast cancer. Surprisingly, the same EVs administered per os enhanced cancer cell invasiveness and metastasis by promoting the epithelial–mesenchymal transition, but this effect diminished after resection of the primary tumor [83]. Similarly, anti-cancer activity associated with the induction of cancer cell apoptosis was shown in the case of oral administration of camel milk EVs to rats with induced breast tumors [84]. Conversely, a single dose of liraglutide-loaded bovine milk-derived EVs effectively reduced blood glucose levels after sublingual administration to diabetic mice, while administration by oral gavage was ineffective [85]. Furthermore, locked nucleic acid-modified antisense oligonucleotides are potent modulators of RNA and protein functions at the molecular level, and thus have recently been proposed for therapeutic applications. In this regard, milk EVs appear to be an efficient shuttle for the oral delivery of such molecules [86].

Additionally, in vitro studies suggest that milk-derived EVs have an affinity for intestinal epithelial cells [87,88,89], and they can be endocytosed due to the presence of glycoproteins on the surface of both EVs and rat/human cells [90,91,92]. Milk EV-induced biological effects may differ depending on the condition of the intestinal epithelium, namely whether it is healthy or cancerous [93], and, in the case of bovine milk EVs, they may depend on the immune status of the donor cow, since dairy cows are classified as low, average or high immune responders [94]. Interestingly, human milk-derived EVs with their miRNA content may be resistant to the unfavorable simulated gastric/pancreatic environment [88,89,95] and are also stable after incubation with saliva or bile juice [89]. Milk EVs administered orally show a wide biodistribution (the signal of labeled EVs is detected in the liver, spleen, kidney, pancreas, ovary, lung, heart, brain and colon), depending on the time of delivery, origin and labeling of EVs [64,83,96,97], and could likely be taken up by tissue-resident macrophages [98].

## 5. Mammalian Cell-Derived EVs—A New Perspective

Notably, some studies have evaluated the possibility of inducing biological effects after oral administration of EVs released by mammalian cells. This may open up new perspectives on the therapeutic application of EVs, since the oral route is noninvasive and thus well-tolerated by patients.

Along these lines, human umbilical cord mesenchymal stem cell-derived EVs, orally administered to both healthy mice and animals suffering from CCl_4_-induced liver failure, accumulated in the liver, which correlated with a dose-dependent inhibition of liver destruction, an antioxidant effect in hepatocytes and a reduction in serum ALT and AST levels. The authors suggested this route of administration because, despite similarities to the intravenous route, it is less invasive [99]. Interestingly, EVs derived from human cardiac stromal cells are more effectively absorbed from the gastrointestinal tract after admixture with casein. Ten minutes after oral ingestion by mdx mice with muscular dystrophy, EVs and casein encapsulated within micelles were found in plasma and in the tibialis, soleus and diaphragm muscles. Moreover, this complex enhanced ejection fraction and skeletal muscle function, which may indicate a possible improvement in dystrophic muscle function [100].

Our recent studies revealed the mechanism of antigen-specific immune tolerance in mice, which is mediated by suppressor T cells [101] that act through the release of EVs carrying immune regulatory miRNA-150 [102,103]. The specificity of EVs’ action is ensured by the surface coating of antibody light chains [104], which also support cell targeting by EVs [105]. As a result, EV-targeted antigen-presenting cells, including macrophages [106], induce immune tolerance to self-antigens and contact allergens [107]. Remarkably, intragastric administration of miRNA-150-transmitting EVs to mice with elicited cutaneous symptoms of delayed-type hypersensitivity to food allergens, i.e., ovalbumin and casein, significantly ameliorated the course of the contact allergic reaction [108,109]. Based on our other research findings [110], we suspect that miRNA-carrying EVs could be endocytosed by macrophages in the digestive tract, which would stimulate these cells to release secondary EVs inducing a tolerogenic effect at distant tissues. Altogether, our findings provide research evidence for the possible application of immune regulatory EVs for oral treatment of various diseases, including allergies and autoimmunity diseases [111]. Moreover, the herein discussed research evidence implies that EVs are applicable for other diseases, as summarized in Table 1.

## 6. Upon Arrival at Destination—EVs in the Intestines

The mammalian intestinal immune system encompasses both the organized lymphoid tissue and scattered cells of innate and adaptive immunity with a substantial regional specialization [112]. Immune cells constantly interact with other intestinal cell populations and the microbiota to maintain the most complex homeostasis within the body, i.e., intestinal homeostasis [113]. These strictly controlled and regulated immune processes could additionally be modulated by food components [114], commensal and pathogenic microbes [115,116], as well as newly established therapeutic strategies [117]. Currently, EVs emerge as crucial factors involved in these immunomodulatory mechanisms [113,118,119], and dysregulation of EV-dependent intercellular signaling is observed in various inflammatory diseases of the gastrointestinal tract [120].

Most absorption of water and nutrients takes place in the intestines. One can speculate that the same could be true for orally delivered EVs since they seem to resist the harsh conditions of the stomach [22]. Accordingly, the digestive stability of milk EV-contained miRNAs has recently been evaluated in vitro by subsequent incubation in three electrolyte and enzyme solutions that mimicked the conditions of oral, gastric and intestinal phases of digestion, respectively. As a result, about 50% of all miRNAs were shown to survive oral and gastric phases of digestion [121]. Furthermore, miRNAs transfected to bovine milk EVs before their oral administration to mice were then detected in various distant tissues [121]. These findings provide indirect evidence for the digestive stability of EVs, which allows them to reach the intestines after oral administration.

According to the abovementioned properties (Table 1), orally-delivered EVs could be considered interesting candidates for therapeutic application to restore intestinal immune homeostasis in various inflammatory disorders. Furthermore, after absorption by the intestine [83], therapeutic EVs could induce expected effects at distant sites. Current observations suggest the involvement of the “neonatal” Fc receptor in the absorption of intact EVs [97] and the role of integrins in both tissue trafficking [122] and subsequent EV uptake by cells [123].

However, before introducing EV-based therapeutics into clinics, several different aspects have to be addressed to fulfill good manufacturing practice requirements. Those include the scalable production process [124], successful reduction in the variability of EVs’ preparations from different biological sources [125], and an increase in their bioavailability and biodistribution [6].

In regard to orally administered EVs, some studies have attempted to evaluate the bioavailability and tissue biodistribution of EVs labeled with fluorochromes in mice after oral gavage and showed vesicle presence in the intestines, liver, spleen, kidney, lung, heart and brain [64]. However, in vivo attempts to estimate the exact efficacy of EVs’ passage through the gastrointestinal tract, their absorption and further biodistribution have encountered some methodological difficulties [126] and are based on the detection of EV-contained miRNAs rather than vesicles themselves [26].

## 7. Conclusions

Although there is still a long road ahead before introducing EVs to clinics [127], growing research evidence seems to prove that orally delivered EVs and EV-transmitted miRNAs may influence immune cells in the digestive tract, which in turn induces effects both locally and in peripheral tissues, likely after transmission via blood and/or lymph (Figure 1, Table 1). Such a possibility would greatly expand the clinical applicability of EVs. However, validation of EV-based oral therapeutics still requires detailed investigation of pharmacokinetics, pharmacodynamics and mechanisms of action.

## Figures and Tables

**Table 1:** Therapeutic effects of orally administered EVs from various sources assessed in animal models of different diseases. AMPK, 5’AMP-activated protein kinase; CCl_4_, carbon tetrachloride; DC, dendritic cell; DSS, dextran sulfate sodium; NF-κB, nuclear factor kappa B; NLRP3, NLR family pyrin domain containing 3.

Disease/Condition	Source of EVs	EVs’ Action	References
DSS-induced colitis	grapefruit	relieving symptoms by inhibiting the production of proinflammatory cytokines	[17]
	broccoli	activation of AMPK in DCs, preventing DC activation and inducing tolerogenic phenotype, reduction in proinflammatory cytokines	[19]
	grape	increasing the proliferation of intestinal stem cells	[20]
	*Akkermansia muciniphila*	decreasing the production of proinflammatory cytokines in the colon and infiltration by inflammatory cells	[43]
	*Escherichia coli* Nissle 1917	ameliorating inflammation in the gut, reducing the increased levels of colonic proinflammatory cytokines, improving intestinal barrier functions	[44]
	*Lactobacillus rhamnosus* GG	reversal of overexpression of NF-κB and NLRP3 signaling pathways involved in inflammation	[45]
	cow and human milk	reducing the histopathological changes and shortening of the colon, reducing the production of proinflammatory cytokines	[70]
tamoxifen-induced ulcerative colitis	bovine milk	improvement of colon condition, macroscopic reduction of colon inflammation	[74]
deoxynivalenol-induced small intestine damage	porcine milk	restoring the disturbed proliferation of intestinal cells, reversal of apoptosis and intercellular junction disorders	[76]
alcohol-induced liver damage	ginger	enhancing the expression of liver detoxifying/antioxidant genes, inhibiting the production of reactive oxygen species	[24]
CCl_4_-induced liver failure	human umbilical cord mesenchymal stem cells	reduction in oxidative stress and apoptosis	[99]
diabetes	*Akkermansia muciniphila*	improving intestinal integrity by regulation of tight junctions through AMPK activation, improving glucose tolerance	[40]
bacterial infections	*Campylobacter jejuni*	immune protection against infection with *C. jejuni*, reducing the bacterial load in cecum	[47]
	*Vibrio cholerae*	strong stimulation of antibody production, long-term protective immune response against *V. cholerae*	[48]
	*Helicobacter pylori*	reducing bacterial numbers, production of *H. pylori*-specific antibodies	[49]
osteoporosis	bovine milk	improving bone mineral density, inhibition of bone resorption, restoration of disturbed intestinal microbiota	[69]
arthritis	bovine milk	reducing proinflammatory cytokines, improved histology of joints	[80]
muscular dystrophy	human cardiac stromal cells	improvement of cardiac ejection fraction, muscle strength and exercise capacity	[100]
solid tumors	cow milk	reducing the primary tumor burden	[83]
	camel milk	induction of cancer cell apoptosis, reduction of angiogenesis and metastasis in tumor tissues	[84]
delayed-type hypersensitivity to food allergens (ovalbumin, casein)	suppressor T cells	suppressing cutaneous symptoms of hypersensitivity reaction	[108,109]

## Data Availability

Not applicable.

## References

[1] Nazimek K., Bryniarski K., Santocki M., Ptak W. (2015). Exosomes as Mediators of Intercellular Communication: Clinical Implications. Pol. Arch. Intern. Med..

[2] Wu P., Zhang B., Ocansey D.K.W., Xu W., Qian H. (2021). Extracellular Vesicles: A Bright Star of Nanomedicine. Biomaterials.

[3] Nazimek K., Bryniarski K. (2020). Perspectives in Manipulating EVs for Therapeutic Applications: Focus on Cancer Treatment. Int. J. Mol. Sci..

[4] Somiya M., Yoshioka Y., Ochiya T. (2017). Division of Molecular and Cellular Medicine, National Cancer Center Research Institute, Tokyo, Japan Drug Delivery Application of Extracellular Vesicles; Insight into Production, Drug Loading, Targeting, and Pharmacokinetics. AIMS Bioeng..

[5] Kim D.H., Kothandan V.K., Kim H.W., Kim K.S., Kim J.Y., Cho H.J., Lee Y., Lee D.-E., Hwang S.R. (2019). Noninvasive Assessment of Exosome Pharmacokinetics In Vivo: A Review. Pharmaceutics.

[6] Kang M., Jordan V., Blenkiron C., Chamley L.W. (2021). Biodistribution of Extracellular Vesicles Following Administration into Animals: A Systematic Review. J. Extracell. Vesicles.

[7] Hunt E.A., Goulding A.M., Deo S.K. (2009). Direct Detection and Quantification of MicroRNAs. Anal. Biochem..

[8] Nelson B.C., Maragh S., Ghiran I.C., Jones J.C., DeRose P.C., Elsheikh E., Vreeland W.N., Wang L. (2020). Measurement and Standardization Challenges for Extracellular Vesicle Therapeutic Delivery Vectors. Nanomedicine.

[9] Wiklander O.P.B., Nordin J.Z., O’Loughlin A., Gustafsson Y., Corso G., Mäger I., Vader P., Lee Y., Sork H., Seow Y. (2015). Extracellular Vesicle in Vivo Biodistribution Is Determined by Cell Source, Route of Administration and Targeting. J. Extracell. Vesicles.

[10] Zhou G., Zhou Y., Chen X. (2017). New Insight into Inter-Kingdom Communication: Horizontal Transfer of Mobile Small RNAs. Front. Microbiol..

[11] Bordoni L., Gabbianelli R. (2021). The Neglected Nutrigenomics of Milk: What Is the Role of Inter-Species Transfer of Small Non-Coding RNA?. Food Biosci..

[12] Tomé-Carneiro J., Fernández-Alonso N., Tomás-Zapico C., Visioli F., Iglesias-Gutierrez E., Dávalos A. (2018). Breast Milk MicroRNAs Harsh Journey towards Potential Effects in Infant Development and Maturation. Lipid Encapsulation Can Help. Pharmacol. Res..

[13] Yu L., Deng Z., Liu L., Zhang W., Wang C. (2020). Plant-Derived Nanovesicles: A Novel Form of Nanomedicine. Front. Bioeng. Biotechnol..

[14] Karamanidou T., Tsouknidas A. (2021). Plant-Derived Extracellular Vesicles as Therapeutic Nanocarriers. Int. J. Mol. Sci..

[15] Urzì O., Raimondo S., Alessandro R. (2021). Extracellular Vesicles from Plants: Current Knowledge and Open Questions. Int. J. Mol. Sci..

[16] Fujita D., Arai T., Komori H., Shirasaki Y., Wakayama T., Nakanishi T., Tamai I. (2018). Apple-Derived Nanoparticles Modulate Expression of Organic-Anion-Transporting Polypeptide (OATP) 2B1 in Caco-2 Cells. Mol. Pharm..

[17] Wang B., Zhuang X., Deng Z.-B., Jiang H., Mu J., Wang Q., Xiang X., Guo H., Zhang L., Dryden G. (2014). Targeted Drug Delivery to Intestinal Macrophages by Bioactive Nanovesicles Released from Grapefruit. Mol. Ther..

[18] Umezu T., Takanashi M., Murakami Y., Ohno S.-I., Kanekura K., Sudo K., Nagamine K., Takeuchi S., Ochiya T., Kuroda M. (2021). Acerola Exosome-like Nanovesicles to Systemically Deliver Nucleic Acid Medicine via Oral Administration. Mol. Ther. Methods Clin. Dev..

[19] Deng Z., Rong Y., Teng Y., Mu J., Zhuang X., Tseng M., Samykutty A., Zhang L., Yan J., Miller D. (2017). Broccoli-Derived Nanoparticle Inhibits Mouse Colitis by Activating Dendritic Cell AMP-Activated Protein Kinase. Mol. Ther..

[20] Ju S., Mu J., Dokland T., Zhuang X., Wang Q., Jiang H., Xiang X., Deng Z.-B., Wang B., Zhang L. (2013). Grape Exosome-like Nanoparticles Induce Intestinal Stem Cells and Protect Mice From DSS-Induced Colitis. Mol. Ther..

[21] Guzman J.R., Conlin V.S., Jobin C. (2013). Diet, Microbiome, and the Intestinal Epithelium: An Essential Triumvirate?. BioMed Res. Int..

[22] Rahimi Ghiasi M., Rahimi E., Amirkhani Z., Salehi R. (2018). Leucine-Rich Repeat-Containing G-Protein Coupled Receptor 5 Gene Overexpression of the Rat Small Intestinal Progenitor Cells in Response to Orally Administered Grape Exosome-like Nanovesicles. Adv. Biomed. Res..

[23] Barker N., Clevers H. (2010). Leucine-Rich Repeat-Containing G-Protein-Coupled Receptors as Markers of Adult Stem Cells. Gastroenterology.

[24] Zhuang X., Deng Z.-B., Mu J., Zhang L., Yan J., Miller D., Feng W., McClain C.J., Zhang H.-G. (2015). Ginger-Derived Nanoparticles Protect against Alcohol-Induced Liver Damage. J. Extracell. Vesicles.

[25] Snow J.W., Hale A.E., Isaacs S.K., Baggish A.L., Chan S.Y. (2013). Ineffective Delivery of Diet-Derived MicroRNAs to Recipient Animal Organisms. RNA Biol..

[26] Liang G., Zhu Y., Sun B., Shao Y., Jing A., Wang J., Xiao Z. (2014). Assessing the Survival of Exogenous Plant MicroRNA in Mice. Food Sci. Nutr..

[27] Chen X., Liu L., Chu Q., Sun S., Wu Y., Tong Z., Fang W., Timko M.P., Fan L. (2021). Large-Scale Identification of Extracellular Plant MiRNAs in Mammals Implicates Their Dietary Intake. PLoS ONE.

[28] Otsuka K., Yamamoto Y., Matsuoka R., Ochiya T. (2018). Maintaining Good MiRNAs in the Body Keeps the Doctor Away?: Perspectives on the Relationship between Food-Derived Natural Products and MicroRNAs in Relation to Exosomes/Extracellular Vesicles. Mol. Nutr. Food Res..

[29] Zempleni J., Baier S.R., Howard K.M., Cui J. (2015). Gene Regulation by Dietary MicroRNAs. Can. J. Physiol. Pharmacol..

[30] Zhang L., Hou D., Chen X., Li D., Zhu L., Zhang Y., Li J., Bian Z., Liang X., Cai X. (2012). Exogenous Plant MIR168a Specifically Targets Mammalian LDLRAP1: Evidence of Cross-Kingdom Regulation by MicroRNA. Cell Res..

[31] Baier S.R., Nguyen C., Xie F., Wood J.R., Zempleni J. (2014). MicroRNAs Are Absorbed in Biologically Meaningful Amounts from Nutritionally Relevant Doses of Cow Milk and Affect Gene Expression in Peripheral Blood Mononuclear Cells, HEK-293 Kidney Cell Cultures, and Mouse Livers. J. Nutr..

[32] Witwer K.W., Zhang C.-Y. (2017). Diet-Derived MicroRNAs: Unicorn or Silver Bullet?. Genes Nutr..

[33] Mar-Aguilar F., Arreola-Triana A., Mata-Cardona D., Gonzalez-Villasana V., Rodríguez-Padilla C., Reséndez-Pérez D. (2020). Evidence of Transfer of MiRNAs from the Diet to the Blood Still Inconclusive. PeerJ.

[34] del Pozo-Acebo L., Lόpez de las Hazas M.C., Margollés A., Dávalos A., García-Ruiz A. (2021). Eating MicroRNAs: Pharmacological Opportunities for Cross-kingdom Regulation and Implications in Host Gene and Gut Microbiota Modulation. Br. J. Pharmacol..

[35] Cavalieri D., Rizzetto L., Tocci N., Rivero D., Asquini E., Si-Ammour A., Bonechi E., Ballerini C., Viola R. (2016). Plant MicroRNAs as Novel Immunomodulatory Agents. Sci. Rep..

[36] Reiner A.T., Somoza V. (2019). Extracellular Vesicles as Vehicles for the Delivery of Food Bioactives. J. Agric. Food Chem..

[37] Cani P.D., de Vos W.M. (2017). Next-Generation Beneficial Microbes: The Case of *Akkermansia Muciniphila*. Front. Microbiol..

[38] Ashrafian F., Shahriary A., Behrouzi A., Moradi H.R., Keshavarz Azizi Raftar S., Lari A., Hadifar S., Yaghoubfar R., Ahmadi Badi S., Khatami S. (2019). *Akkermansia Muciniphila*-Derived Extracellular Vesicles as a Mucosal Delivery Vector for Amelioration of Obesity in Mice. Front. Microbiol..

[39] Yaghoubfar R., Behrouzi A., Ashrafian F., Shahryari A., Moradi H.R., Choopani S., Hadifar S., Vaziri F., Nojoumi S.A., Fateh A. (2020). Modulation of Serotonin Signaling/Metabolism by *Akkermansia Muciniphila* and Its Extracellular Vesicles through the Gut-Brain Axis in Mice. Sci. Rep..

[40] Chelakkot C., Choi Y., Kim D.-K., Park H.T., Ghim J., Kwon Y., Jeon J., Kim M.-S., Jee Y.-K., Gho Y.S. (2018). *Akkermansia Muciniphila*-Derived Extracellular Vesicles Influence Gut Permeability through the Regulation of Tight Junctions. Exp. Mol. Med..

[41] Ashrafian F., Behrouzi A., Shahriary A., Ahmadi Badi S., Davari M., Khatami S., Rahimi Jamnani F., Fateh A., Vaziri F., Siadat S.D. (2019). Comparative Study of Effect of *Akkermansia Muciniphila* and Its Extracellular Vesicles on Toll-like Receptors and Tight Junction. Gastroenterol. Hepatol. Bed Bench.

[42] Lee K.-E., Kim J.-K., Han S.-K., Lee D.Y., Lee H.-J., Yim S.-V., Kim D.-H. (2020). The Extracellular Vesicle of Gut Microbial *Paenalcaligenes Hominis* Is a Risk Factor for Vagus Nerve-Mediated Cognitive Impairment. Microbiome.

[43] Kang C.-S., Ban M., Choi E.-J., Moon H.-G., Jeon J.-S., Kim D.-K., Park S.-K., Jeon S.G., Roh T.-Y., Myung S.-J. (2013). Extracellular Vesicles Derived from Gut Microbiota, Especially *Akkermansia Muciniphila*, Protect the Progression of Dextran Sulfate Sodium-Induced Colitis. PLoS ONE.

[44] Fábrega M.-J., Rodríguez-Nogales A., Garrido-Mesa J., Algieri F., Badía J., Giménez R., Gálvez J., Baldomà L. (2017). Intestinal Anti-Inflammatory Effects of Outer Membrane Vesicles from *Escherichia Coli* Nissle 1917 in DSS-Experimental Colitis in Mice. Front. Microbiol..

[45] Tong L., Zhang X., Hao H., Liu Q., Zhou Z., Liang X., Liu T., Gong P., Zhang L., Zhai Z. (2021). *Lactobacillus Rhamnosus* GG Derived Extracellular Vesicles Modulate Gut Microbiota and Attenuate Inflammatory in DSS-Induced Colitis Mice. Nutrients.

[46] Kaparakis M., Turnbull L., Carneiro L., Firth S., Coleman H.A., Parkington H.C., Le Bourhis L., Karrar A., Viala J., Mak J. (2010). Bacterial Membrane Vesicles Deliver Peptidoglycan to NOD1 in Epithelial Cells. Cell. Microbiol..

[47] Singh A., Khan A., Ghosh T., Mondal S., Mallick A.I. (2021). Gut Microbe-Derived Outer Membrane Vesicles: A Potential Platform to Control Cecal Load of *Campylobacter jejuni*. ACS Infect. Dis..

[48] Schild S., Nelson E.J., Camilli A. (2008). Immunization with *Vibrio cholerae* Outer Membrane Vesicles Induces Protective Immunity in Mice. Infect. Immun..

[49] Keenan J., Oliaro J., Domigan N., Potter H., Aitken G., Allardyce R., Roake J. (2000). Immune Response to an 18-Kilodalton Outer Membrane Antigen Identifies Lipoprotein 20 as a *Helicobacter pylori* Vaccine Candidate. Infect. Immun..

[50] Jiang X., You L., Zhang Z., Cui X., Zhong H., Sun X., Ji C., Chi X. (2021). Biological Properties of Milk-Derived Extracellular Vesicles and Their Physiological Functions in Infant. Front. Cell Dev. Biol..

[51] Feng X., Chen X., Zheng X., Zhu H., Qi Q., Liu S., Zhang H., Che J. (2021). Latest Trend of Milk Derived Exosomes: Cargos, Functions, and Applications. Front. Nutr..

[52] Admyre C., Johansson S.M., Qazi K.R., Filén J.-J., Lahesmaa R., Norman M., Neve E.P.A., Scheynius A., Gabrielsson S. (2007). Exosomes with Immune Modulatory Features Are Present in Human Breast Milk. J. Immunol..

[53] Zhou Q., Li M., Wang X., Li Q., Wang T., Zhu Q., Zhou X., Wang X., Gao X., Li X. (2012). Immune-Related MicroRNAs Are Abundant in Breast Milk Exosomes. Int. J. Biol. Sci..

[54] Gu Y., Li M., Wang T., Liang Y., Zhong Z., Wang X., Zhou Q., Chen L., Lang Q., He Z. (2012). Lactation-Related MicroRNA Expression Profiles of Porcine Breast Milk Exosomes. PLoS ONE.

[55] Sun Q., Chen X., Yu J., Zen K., Zhang C.-Y., Li L. (2013). Immune Modulatory Function of Abundant Immune-Related MicroRNAs in Microvesicles from Bovine Colostrum. Protein Cell.

[56] van Herwijnen M.J.C., Driedonks T.A.P., Snoek B.L., Kroon A.M.T., Kleinjan M., Jorritsma R., Pieterse C.M.J., Nolte-’t Hoen E.N.M., Wauben M.H.M. (2018). Abundantly Present MiRNAs in Milk-Derived Extracellular Vesicles Are Conserved Between Mammals. Front. Nutr..

[57] Lin D., Chen T., Xie M., Li M., Zeng B., Sun R., Zhu Y., Ye D., Wu J., Sun J. (2020). Oral Administration of Bovine and Porcine Milk Exosome Alter MiRNAs Profiles in Piglet Serum. Sci. Rep..

[58] Lukasik A., Zielenkiewicz P. (2014). In Silico Identification of Plant MiRNAs in Mammalian Breast Milk Exosomes–A Small Step Forward?. PLoS ONE.

[59] Kirchner B., Buschmann D., Paul V., Pfaffl M.W. (2020). Postprandial Transfer of Colostral Extracellular Vesicles and Their Protein and MiRNA Cargo in Neonatal Calves. PLoS ONE.

[60] Auerbach A., Vyas G., Li A., Halushka M., Witwer K. (2016). Uptake of Dietary Milk MiRNAs by Adult Humans: A Validation Study. F1000Research.

[61] Sadri M., Shu J., Kachman S.D., Cui J., Zempleni J. (2020). Milk Exosomes and MiRNA Cross the Placenta and Promote Embryo Survival in Mice. Reproduction.

[62] Leiferman A., Shu J., Grove R., Cui J., Adamec J., Zempleni J. (2018). A Diet Defined by Its Content of Bovine Milk Exosomes and Their RNA Cargos Has Moderate Effects on Gene Expression, Amino Acid Profiles and Grip Strength in Skeletal Muscle in C57BL/6 Mice. J. Nutr. Biochem..

[63] Go G., Jeon J., Lee G., Lee J.H., Lee S.H. (2021). Bovine Milk Extracellular Vesicles Induce the Proliferation and Differentiation of Osteoblasts and Promote Osteogenesis in Rats. J. Food Biochem..

[64] Manca S., Upadhyaya B., Mutai E., Desaulniers A.T., Cederberg R.A., White B.R., Zempleni J. (2018). Milk Exosomes Are Bioavailable and Distinct MicroRNA Cargos Have Unique Tissue Distribution Patterns. Sci. Rep..

[65] Parry H.A., Mobley C.B., Mumford P.W., Romero M.A., Haun C.T., Zhang Y., Roberson P.A., Zempleni J., Ferrando A.A., Vechetti I.J. (2019). Bovine Milk Extracellular Vesicles (EVs) Modification Elicits Skeletal Muscle Growth in Rats. Front. Physiol..

[66] Zhou F., Paz H.A., Sadri M., Cui J., Kachman S.D., Fernando S.C., Zempleni J. (2019). Dietary Bovine Milk Exosomes Elicit Changes in Bacterial Communities in C57BL/6 Mice. Am. J. Physiol.-Gastrointest. Liver Physiol..

[67] Du C., Quan S., Nan X., Zhao Y., Shi F., Luo Q., Xiong B. (2021). Effects of Oral Milk Extracellular Vesicles on the Gut Microbiome and Serum Metabolome in Mice. Food Funct..

[68] Tong L., Hao H., Zhang X., Zhang Z., Lv Y., Zhang L., Yi H. (2020). Oral Administration of Bovine Milk-Derived Extracellular Vesicles Alters the Gut Microbiota and Enhances Intestinal Immunity in Mice. Mol. Nutr. Food Res..

[69] Yun B., Maburutse B.E., Kang M., Park M.R., Park D.J., Kim Y., Oh S. (2020). Short Communication: Dietary Bovine Milk-Derived Exosomes Improve Bone Health in an Osteoporosis-Induced Mouse Model. J. Dairy Sci..

[70] Reif S., Elbaum-Shiff Y., Koroukhov N., Shilo I., Musseri M., Golan-Gerstl R. (2020). Cow and Human Milk-Derived Exosomes Ameliorate Colitis in DSS Murine Model. Nutrients.

[71] Roefs M.T., Sluijter J.P.G., Vader P. (2020). Extracellular Vesicle-Associated Proteins in Tissue Repair. Trends Cell Biol..

[72] Shelke G.V., Yin Y., Jang S.C., Lässer C., Wennmalm S., Hoffmann H.J., Li L., Gho Y.S., Nilsson J.A., Lötvall J. (2019). Endosomal Signalling via Exosome Surface TGFβ-1. J. Extracell. Vesicles.

[73] Pieters B.C.H., Arntz O.J., Bennink M.B., Broeren M.G.A., van Caam A.P.M., Koenders M.I., van Lent P.L.E.M., van den Berg W.B., de Vries M., van der Kraan P.M. (2015). Commercial Cow Milk Contains Physically Stable Extracellular Vesicles Expressing Immunoregulatory TGF-β. PLoS ONE.

[74] Stremmel W., Weiskirchen R., Melnik B.C. (2020). Milk Exosomes Prevent Intestinal Inflammation in a Genetic Mouse Model of Ulcerative Colitis: A Pilot Experiment. Inflamm. Intest. Dis..

[75] Wu D., Kittana H., Shu J., Kachman S.D., Cui J., Ramer-Tait A.E., Zempleni J. (2019). Dietary Depletion of Milk Exosomes and Their MicroRNA Cargos Elicits a Depletion of MiR-200a-3p and Elevated Intestinal Inflammation and CXCL9 Expression in Mdr1a−/- Mice. Curr. Dev. Nutr..

[76] Xie M.-Y., Chen T., Xi Q.-Y., Hou L.-J., Luo J.-Y., Zeng B., Li M., Sun J.-J., Zhang Y.-L. (2020). Porcine Milk Exosome MiRNAs Protect Intestinal Epithelial Cells against Deoxynivalenol-Induced Damage. Biochem. Pharmacol..

[77] Chen T., Xie M.-Y., Sun J.-J., Ye R.-S., Cheng X., Sun R.-P., Wei L.-M., Li M., Lin D.-L., Jiang Q.-Y. (2016). Porcine Milk-Derived Exosomes Promote Proliferation of Intestinal Epithelial Cells. Sci. Rep..

[78] Dong P., Zhang Y., Yan D., Wang Y., Xu X., Zhao Y., Xiao T. (2020). Protective Effects of Human Milk-Derived Exosomes on Intestinal Stem Cells Damaged by Oxidative Stress. Cell Transplant..

[79] Hock A., Miyake H., Li B., Lee C., Ermini L., Koike Y., Chen Y., Määttänen P., Zani A., Pierro A. (2017). Breast Milk-Derived Exosomes Promote Intestinal Epithelial Cell Growth. J. Pediatr. Surg..

[80] Arntz O.J., Pieters B.C.H., Oliveira M.C., Broeren M.G.A., Bennink M.B., de Vries M., van Lent P.L.E.M., Koenders M.I., van den Berg W.B., van der Kraan P.M. (2015). Oral Administration of Bovine Milk Derived Extracellular Vesicles Attenuates Arthritis in Two Mouse Models. Mol. Nutr. Food Res..

[81] del Pozo-Acebo L., López de las Hazas M.-C., Tomé-Carneiro J., Gil-Cabrerizo P., San-Cristobal R., Busto R., García-Ruiz A., Dávalos A. (2021). Bovine Milk-Derived Exosomes as a Drug Delivery Vehicle for MiRNA-Based Therapy. Int. J. Mol. Sci..

[82] Agrawal A.K., Aqil F., Jeyabalan J., Spencer W.A., Beck J., Gachuki B.W., Alhakeem S.S., Oben K., Munagala R., Bondada S. (2017). Milk-Derived Exosomes for Oral Delivery of Paclitaxel. Nanomedicine.

[83] Samuel M., Fonseka P., Sanwlani R., Gangoda L., Chee S.H., Keerthikumar S., Spurling A., Chitti S.V., Zanker D., Ang C.-S. (2021). Oral Administration of Bovine Milk-Derived Extracellular Vesicles Induces Senescence in the Primary Tumor but Accelerates Cancer Metastasis. Nat. Commun..

[84] Badawy A.A., El-Magd M.A., AlSadrah S.A. (2018). Therapeutic Effect of Camel Milk and Its Exosomes on MCF7 Cells In Vitro and In Vivo. Integr. Cancer Ther..

[85] Xu M., Chen G., Dong Y., Yang J., Liu Y., Song H., Song H., Wang Y. (2022). Liraglutide-Loaded Milk Exosomes Lower Blood Glucose When Given by Sublingual Route. ChemMedChem.

[86] Grossen P., Portmann M., Koller E., Duschmalé M., Minz T., Sewing S., Pandya N.J., van Geijtenbeek S.K., Ducret A., Kusznir E.-A. (2021). Evaluation of Bovine Milk Extracellular Vesicles for the Delivery of Locked Nucleic Acid Antisense Oligonucleotides. Eur. J. Pharm. Biopharm..

[87] Carobolante G., Mantaj J., Ferrari E., Vllasaliu D. (2020). Cow Milk and Intestinal Epithelial Cell-Derived Extracellular Vesicles as Systems for Enhancing Oral Drug Delivery. Pharmaceutics.

[88] Liao Y., Du X., Li J., Lönnerdal B. (2017). Human Milk Exosomes and Their MicroRNAs Survive Digestion in Vitro and Are Taken up by Human Intestinal Cells. Mol. Nutr. Food Res..

[89] Rani P., Vashisht M., Golla N., Shandilya S., Onteru S.K., Singh D. (2017). Milk MiRNAs Encapsulated in Exosomes Are Stable to Human Digestion and Permeable to Intestinal Barrier in Vitro. J. Funct. Foods.

[90] Sukreet S., Zhang H., Adamec J., Cui J., Zempleni J. (2017). Identification of Glycoproteins on the Surface of Bovine Milk Exosomes and Intestinal Cells That Facilitate Exosome Uptake in Human Colon Carcinoma Caco-2 Cells. FASEB J..

[91] Kusuma R.J., Manca S., Friemel T., Sukreet S., Nguyen C., Zempleni J. (2016). Human Vascular Endothelial Cells Transport Foreign Exosomes from Cow’s Milk by Endocytosis. Am. J. Physiol.-Cell Physiol..

[92] Wolf T., Baier S.R., Zempleni J. (2015). The Intestinal Transport of Bovine Milk Exosomes Is Mediated by Endocytosis in Human Colon Carcinoma Caco-2 Cells and Rat Small Intestinal IEC-6 Cells. J. Nutr..

[93] Reif S., Elbaum Shiff Y., Golan-Gerstl R. (2019). Milk-Derived Exosomes (MDEs) Have a Different Biological Effect on Normal Fetal Colon Epithelial Cells Compared to Colon Tumor Cells in a MiRNA-Dependent Manner. J. Transl. Med..

[94] Ross M., Atalla H., Karrow N., Mallard B.A. (2021). The Bioactivity of Colostrum and Milk Exosomes of High, Average, and Low Immune Responder Cows on Human Intestinal Epithelial Cells. J. Dairy Sci..

[95] Izumi H., Kosaka N., Shimizu T., Sekine K., Ochiya T., Takase M. (2012). Bovine Milk Contains MicroRNA and Messenger RNA that are Stable under Degradative Conditions. J. Dairy Sci..

[96] Munagala R., Aqil F., Jeyabalan J., Gupta R.C. (2016). Bovine Milk-Derived Exosomes for Drug Delivery. Cancer Lett..

[97] Betker J.L., Angle B.M., Graner M.W., Anchordoquy T.J. (2019). The Potential of Exosomes From Cow Milk for Oral Delivery. J. Pharm. Sci..

[98] Izumi H., Tsuda M., Sato Y., Kosaka N., Ochiya T., Iwamoto H., Namba K., Takeda Y. (2015). Bovine Milk Exosomes Contain MicroRNA and MRNA and Are Taken up by Human Macrophages. J. Dairy Sci..

[99] Yan Y., Jiang W., Tan Y., Zou S., Zhang H., Mao F., Gong A., Qian H., Xu W. (2017). HucMSC Exosome-Derived GPX1 is Required for the Recovery of Hepatic Oxidant Injury. Mol. Ther..

[100] Aminzadeh M.A., Fournier M., Akhmerov A., Jones-Ungerleider K.C., Valle J.B., Marbán E. (2021). Casein-Enhanced Uptake and Disease-Modifying Bioactivity of Ingested Extracellular Vesicles. J. Extracell. Vesicles.

[101] Ptak W., Nazimek K., Askenase P.W., Bryniarski K. (2015). From Mysterious Supernatant Entity to MiRNA-150 in Antigen-Specific Exosomes: A History of Hapten-Specific T Suppressor Factor. Arch. Immunol. Ther. Exp..

[102] Bryniarski K., Ptak W., Jayakumar A., Püllmann K., Caplan M.J., Chairoungdua A., Lu J., Adams B.D., Sikora E., Nazimek K. (2013). Antigen-Specific, Antibody-Coated, Exosome-like Nanovesicles Deliver Suppressor T-Cell MicroRNA-150 to Effector T Cells to Inhibit Contact Sensitivity. J. Allergy Clin. Immunol..

[103] Bryniarski K., Ptak W., Martin E., Nazimek K., Szczepanik M., Sanak M., Askenase P.W. (2015). Free Extracellular MiRNA Functionally Targets Cells by Transfecting Exosomes from Their Companion Cells. PLoS ONE.

[104] Nazimek K., Askenase P.W., Bryniarski K. (2018). Antibody Light Chains Dictate the Specificity of Contact Hypersensitivity Effector Cell Suppression Mediated by Exosomes. Int. J. Mol. Sci..

[105] Nazimek K., Bryniarski K. (2021). Increasing the Therapeutic Efficacy of Extracellular Vesicles From the Antigen-Specific Antibody and Light Chain Perspective. Front. Cell Dev. Biol..

[106] Nazimek K., Ptak W., Nowak B., Ptak M., Askenase P.W., Bryniarski K. (2015). Macrophages Play an Essential Role in Antigen-Specific Immune Suppression Mediated by T CD8^+^ Cell-Derived Exosomes. Immunology.

[107] Nazimek K., Bustos-Morán E., Blas-Rus N., Nowak B., Ptak W., Askenase P.W., Sánchez-Madrid F., Bryniarski K. (2019). Syngeneic Red Blood Cell-Induced Extracellular Vesicles Suppress Delayed-Type Hypersensitivity to Self-Antigens in Mice. Clin. Exp. Allergy.

[108] Nazimek K., Bryniarski K., Ptak W., Groot Kormelink T., Askenase P.W. (2020). Orally Administered Exosomes Suppress Mouse Delayed-Type Hypersensitivity by Delivering MiRNA-150 to Antigen-Primed Macrophage APC Targeted by Exosome-Surface Anti-Peptide Antibody Light Chains. Int. J. Mol. Sci..

[109] Wąsik M., Nazimek K., Nowak B., Askenase P.W., Bryniarski K. (2019). Delayed-Type Hypersensitivity Underlying Casein Allergy is Suppressed by Extracellular Vesicles Carrying MiRNA-150. Nutrients.

[110] Nazimek K., Bustos-Morán E., Blas-Rus N., Nowak B., Totoń-Żurańska J., Seweryn M.T., Wołkow P., Woźnicka O., Szatanek R., Siedlar M. (2021). Antibodies Enhance the Suppressive Activity of Extracellular Vesicles in Mouse Delayed-Type Hypersensitivity. Pharmaceuticals.

[111] Nazimek K., Bryniarski K. (2020). Approaches to Inducing Antigen-Specific Immune Tolerance in Allergy and Autoimmunity: Focus on Antigen-Presenting Cells and Extracellular Vesicles. Scand. J. Immunol..

[112] Mowat A.M., Agace W.W. (2014). Regional Specialization within the Intestinal Immune System. Nat. Rev. Immunol..

[113] Chang X., Wang S.-L., Zhao S.-B., Shi Y.-H., Pan P., Gu L., Yao J., Li Z.-S., Bai Y. (2020). Extracellular Vesicles with Possible Roles in Gut Intestinal Tract Homeostasis and IBD. Mediat. Inflamm..

[114] Hachimura S., Totsuka M., Hosono A. (2018). Immunomodulation by Food: Impact on Gut Immunity and Immune Cell Function. Biosci. Biotechnol. Biochem..

[115] Cieza R.J., Cao A.T., Cong Y., Torres A.G. (2012). Immunomodulation for Gastrointestinal Infections. Expert Rev. Anti-Infect. Ther..

[116] Kayama H., Takeda K. (2012). Regulation of Intestinal Homeostasis by Innate and Adaptive Immunity. Int. Immunol..

[117] Lee Y., Kamada N., Moon J.J. (2021). Oral Nanomedicine for Modulating Immunity, Intestinal Barrier Functions, and Gut Microbiome. Adv. Drug Deliv. Rev..

[118] Shen Q., Huang Z., Yao J., Jin Y. (2022). Extracellular Vesicles-Mediated Interaction within Intestinal Microenvironment in Inflammatory Bowel Disease. J. Adv. Res..

[119] Diaz-Garrido N., Cordero C., Olivo-Martinez Y., Badia J., Baldomà L. (2021). Cell-to-Cell Communication by Host-Released Extracellular Vesicles in the Gut: Implications in Health and Disease. Int. J. Mol. Sci..

[120] Filip R. (2021). An Update on the Role of Extracellular Vesicles in the Pathogenesis of Necrotizing Enterocolitis and Inflammatory Bowel Diseases. Cells.

[121] López de las Hazas M.-C., del Pozo-Acebo L., Hansen M.S., Gil-Zamorano J., Mantilla-Escalante D.C., Gómez-Coronado D., Marín F., Garcia-Ruiz A., Rasmussen J.T., Dávalos A. (2022). Dietary Bovine Milk MiRNAs Transported in Extracellular Vesicles are Partially Stable during GI Digestion, are Bioavailable and Reach Target Tissues but Need a Minimum Dose to Impact on Gene Expression. Eur. J. Nutr..

[122] Charoenviriyakul C., Takahashi Y., Morishita M., Nishikawa M., Takakura Y. (2018). Role of Extracellular Vesicle Surface Proteins in the Pharmacokinetics of Extracellular Vesicles. Mol. Pharm..

[123] Nolte M.A., Nolte-’t Hoen E.N.M., Margadant C. (2021). Integrins Control Vesicular Trafficking; New Tricks for Old Dogs. Trends Biochem. Sci..

[124] Staubach S., Bauer F.N., Tertel T., Börger V., Stambouli O., Salzig D., Giebel B. (2021). Scaled Preparation of Extracellular Vesicles from Conditioned Media. Adv. Drug Deliv. Rev..

[125] Gandham S., Su X., Wood J., Nocera A.L., Alli S.C., Milane L., Zimmerman A., Amiji M., Ivanov A.R. (2020). Technologies and Standardization in Research on Extracellular Vesicles. Trends Biotechnol..

[126] Akuma P., Okagu O.D., Udenigwe C.C. (2019). Naturally Occurring Exosome Vesicles as Potential Delivery Vehicle for Bioactive Compounds. Front. Sustain. Food Syst..

[127] Aarts J., Boleij A., Pieters B.C.H., Feitsma A.L., van Neerven R.J.J., ten Klooster J.P., M’Rabet L., Arntz O.J., Koenders M.I., van de Loo F.A.J. (2021). Flood Control: How Milk-Derived Extracellular Vesicles Can Help to Improve the Intestinal Barrier Function and Break the Gut–Joint Axis in Rheumatoid Arthritis. Front. Immunol..

